# Exploring the Impact of Tinnitus on Work Productivity

**DOI:** 10.3390/brainsci16020150

**Published:** 2026-01-29

**Authors:** Eldre Beukes, Jennine A. Sharpe, Gerhard Andersson, Vinaya Manchaiah

**Affiliations:** 1 Centre for Better Living, Anglia Ruskin University, Cambridge CB1 1TP, UK; jas313@cam.ac.uk; 2 Virtual Hearing Lab, Collaborative Initiative Between the University of Colorado and the University of Pretoria, Aurora, CO 80045, USA; vinaya.manchaiah@cuanschutz.edu; 3 Department of Material Sciences and Metallurgy, University of Cambridge, Cambridge CB3 0FS, UK; 4 Department of Behavioural Sciences and Learning, Department of Biomedical and Clinical Sciences, Linköping University, SE-581 83 Linköping, Sweden; gerhard.andersson@liu.se; 5 Department of Clinical Neuroscience, Karolinska Institutet, SE-171 77 Stockholm, Sweden; 6 Department of Health, Education and Technology, Luleå University of Technology, SE-971 87 Luleå, Sweden; 7 HEI-Lab: Digital Human-Environment Interaction Labs, Department of Otolaryngology-Head and Neck Surgery, Lusófona University, 1749-024 Lisbon, Portugal; 8 Department of Otolaryngology-Head and Neck Surgery, University of Colorado School of Medicine, Aurora, CO 80045, USA; 9 UCHealth Hearing and Balance, University of Colorado Hospital, Aurora, CO 80045, USA; 10 Department of Speech-Language Pathology and Audiology, University of Pretoria, Pretoria 0002, South Africa; 11 Department of Speech and Hearing, School of Allied Health Sciences, Manipal University, Manipal 576104, India

**Keywords:** tinnitus, workplace functioning, productivity, cognitive load, intervention, occupational health

## Abstract

**Background/Objectives**: Tinnitus affects various aspects of health, yet its impact on occupational functioning remains less well explored. This study investigated the impact of tinnitus on work productivity, the role of comorbidities of anxiety, depression, and quality of life, and explored whether an evidence-based intervention may potentially influence workplace functioning. **Methods**: The study used cross-sectional and longitudinal data and employed mixed methods design to enable comparing work productivity before and after undertaking ICBT for tinnitus. Data were collected from 449 participants (64% employed; mean age of 54.4 years) who were seeking to enroll in an internet-based psychological intervention. Of these, 200 also completed an internet-based cognitive behavioral therapy intervention (ICBT). Data were analyzed using descriptive statistics, analysis of variance, qualitative content analysis and comparisons made between those whose work was unaffected due to tinnitus and those who worked less. **Results**: Pre-intervention, 81% reported no change in work patterns. Because of tinnitus 11% worked fewer hours, 7% had stopped working, and 1% received disability allowance. Participants were significantly less effective in their work capacity prior to undertaking the intervention compared to post-intervention and at follow-up; for the overall sample and post-intervention improvements were observed in tinnitus distress, anxiety, depression, and insomnia. There was significant time difference between group interactions for tinnitus, anxiety and insomnia but not for depression and quality of life when comparing those working and not working. Qualitative findings highlighted challenges related to attention, fatigue, and communication, as well as coping strategies and role modifications. **Conclusions**: Further studies are required to build on this exploratory research. The present findings underscore the need for workplace policies recognizing tinnitus as a potential disability and providing reasonable accommodation and options to access tinnitus interventions. Clinical interventions should also consider how to improve workplace functioning in individuals with bothersome tinnitus. Future research should explore ways to address cognitive load and enhance productivity.

## 1. Introduction

Strong economies rely on high productivity and employment rates. Factors such as absenteeism and reduced work performance can negatively impact economic growth [1]. Identifying strategies to enhance productivity is therefore essential and requires multifaceted approaches. This may involve protocols and policies to accommodate health-related difficulties. One potentially overlooked factor is the contribution of tinnitus to workplace functioning and productivity.

Tinnitus is defined as the conscious perception of sound in the absence of an external auditory stimulus. Its prevalence is estimated at 15%, although only a subset of individuals experiences bothersome tinnitus that interferes with daily functioning [2]. The primary risk factors for tinnitus include hearing loss and noise exposure, with occupations such as the military, construction and manufacturing involving high noise levels, typically above 85 dBA [3]. Despite this association, the impact of tinnitus on work ability has received limited attention.

Tinnitus symptoms may be exacerbated in quiet environments, during stressful or emotionally demanding situations, when tasks require sustained concentration, and during social interactions or sudden changes in sound environments [4]. These conditions are common in workplace settings, suggesting that tinnitus could significantly affect occupational functioning and may lead to earlier retirement [5]. Having tinnitus and hyperacusis predicted reduced work capacity and hyperacusis predicted work absence [6]. Furthermore, hearing- and tinnitus-related difficulties were predictors of long-term work incapacity [7]. Certain noisy occupations are associated with an increased risk of bothersome tinnitus, and hence more likely to lead to a reduction in work capacity [8] particularly for military veterans [9]. It has furthermore been found that for those on a disability pension for hearing loss, vertigo or tinnitus, there is a 14% increased risk of mortality [10], making finding ways to reduce the impact of these disabilities important. There are thus indications that tinnitus can affect work productivity. What is not clear is if there are any interventions that can help minimize the impact of tinnitus. There were for instance no identified studies that have examined the effect of tinnitus interventions on workplace productivity.

Cognitive mechanisms may further explain reduced productivity. Tinnitus imposes an additional cognitive load, competing for limited working memory resources, particularly under demanding conditions [11]. Possible mechanisms include those associated with hyperactivity in the fronto-parietal attention networks resulting in delayed attentional disengagement and poorer updating of working memory buffers [12]. Consequently, individuals with tinnitus demonstrate slower reaction times and poorer performance on attention-switching tasks compared to those without tinnitus [13]. Comorbidities such as hearing loss, insomnia and anxiety may compound these effects, further impairing performance on tasks requiring high attentional control and reducing cognitive capacity [14]. Although these links have been made, there is still a lack of clarity regarding the impact tinnitus has on functioning at work and whether functioning can be improved.

Although evidence-based interventions such as cognitive behavioral therapy (CBT) have been shown to reduce tinnitus distress and associated comorbidities [15], little is known about whether such interventions improve workplace functioning. To address this gap, the present study aimed to quantify the impact of tinnitus on work productivity and examine whether these effects can be mitigated through intervention. Specifically, the following research questions were explored: (i) What impact does tinnitus have on the ability to work? (ii) Do comorbidities such as hearing loss and depression further reduce work productivity? and (iii) Can an evidence-based tinnitus intervention improve workplace functioning?

## 2. Materials and Methods

### 2.1. Study Design

The study used a mixed methods design and was nested in clinical trials (Clinical Trials.gov registration numbers NCT04004260, NCT04335812) that were aimed at evaluating the efficacy of internet-based cognitive behavioral therapy (ICBT) for tinnitus [15,16]. Ethics approval was granted by the Institutional Review Board at Lamar University (IRB-FY17-209, approved on 7 June 2019 and IRB-FY20-200 approved on 2 April 2000). All participants completed informed consent prior to participating in the study.

### 2.2. Participants

Both cross-sectional and longitudinal data (pre-intervention, post-intervention and at 2-month follow-up) were used based on the pooled data from the studies. Change in workplace productivity after intervention was assessed but has not been reported before. All participants (*n* = 449) who were enrolled in the ICBT clinical trials were asked to complete a series of questionnaires before and after the intervention through the online platform (i.e., iTerapi) that was used to administer ICBT. The inclusion (i.e., adults > 18 years, access to computer, having tinnitus for longer than 3 months, at least a mild severity of tinnitus) and exclusion criteria (i.e., having significant depression, psychiatric condition, pulsatile or objective tinnitus, currently undergoing other tinnitus therapies) are detailed in the clinical trial publications [16,17,18]. Chronic tinnitus was defined as experiencing tinnitus for at least 3 months or longer.

### 2.3. Data Collection

Data collection took place at three time points, pre-intervention, post-intervention and two-month follow-up. The baseline questionnaire included detailed demographic and hearing-related questions. In addition, various structured and unstructured questionnaires were administered at all three time points as detailed in the clinical trials reporting [16,17,18].

The following work-related questions and some general measures were used in this study.

- Do you work less due to having tinnitus? (No or yes. If yes, select reduced hours, stopped working, disability allowance?).
- Has the tinnitus experience made you less effective at doing your work or daily tasks? (No, slightly, considerable, very much so).
- The Tinnitus Functional Index (TFI) [19] to measure the degree of tinnitus distress.
- To assess common comorbidities associated with tinnitus the following questionnaires were selected. To assess anxiety, the Generalized Anxiety Disorder Questionnaire (GAD7) [20]. To assess depression, the Patient Health Questionnaire 9; (PHQ9) [21]. The insomnia-severity index (ISI) [22] and health-related quality of life (Euroqol EQ-5D-5L), [23] were also included.
- Open-ended question: What describes your work (select from entry level or unskilled work, skilled/professional work, skilled or professional work, retired, not working)

From question (i) the study sample was categorized into two subgroups based on whether their work was unaffected by tinnitus (answering no) or affected resulting in working reduced hours (answered yes) to compare the response.

### 2.4. ICBT Intervention

The ICBT intervention was designed to combine psychoeducation, relaxation training, cognitive behavioral therapy strategies, and behavioral activation to enable long-term self-management of tinnitus [24]. To improve accessibility it was delivered on a secure web platform, for which participants used a password-protected login. The intervention was undertaken over an 8-week period. During this time, 2–3 modules were shared per week as outlined in Table 1. Modules contained a mixture of information, videos, quizzes, diagrams, suggested techniques to apply to daily life, worksheets to keep track of progress, solutions for common problems, and downloadable information. Audiological guidance was provided by an experienced tinnitus clinician (EB) together with supervision from a licensed psychologist (GA). Guidance aimed to support individuals who participated in the intervention via a two-way messaging system within the e-Platform. Guidance included monitoring progress, monitoring weekly scores, providing weekly feedback on worksheets completed, outlining the content of new modules, and answering questions.

### 2.5. Data Analysis

Quantitative statistical analysis was done using Jamovi (version 2.3.28.0). Descriptive statistics included age, gender, ethnicity, race, and tinnitus characteristics. Chi-square analysis and *t*-tests were used to compare group differences. Clinical variables were tinnitus distress, anxiety, depression and quality of life. Repeated measures ANOVAs were run and Bonferroni corrected post hoc testing between the groups when needed. Where the data did not fit parametric assumptions, non-parametric testing was performed. Response to open-ended questions were coded using qualitative content analysis [25] using NVivo software Version 15.

## 3. Results

### 3.1. Clinical Characteristics

There were 449 participants completing the questionnaire, although some questions were not answered by all participants. Of these, the majority were not working less (*n* = 285, 63%). Of those not working or working reduced hours (*n* = 164, 27%), 126 (77%) had retired and 38 (23%) stopped working prior to retirement. There were significant gender differences as there were more females who were not working than males. There were also significantly more Hispanic participants working but no differences in race representation. There were no group differences regarding tinnitus duration as shown in Table 2.

Comparisons were made between those completing the intervention and those not, as seen in Table 3. Overall, there were no significant group differences except that there were more males in the not completing group and more females in those completing the intervention and post-intervention questionnaires. Participants indicating that tinnitus was the reason for working less at pre-intervention, post-intervention and at follow-up is seen in Table 4.

**Figure 1:**
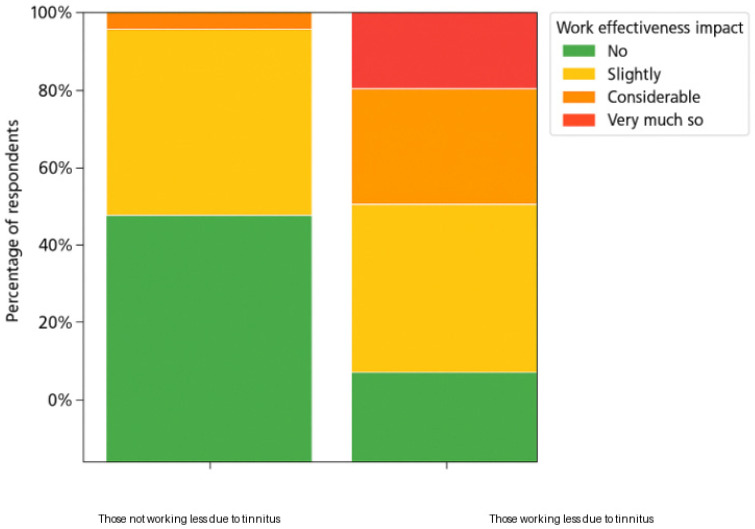
Comparing the impact of tinnitus on work productivity ratings for those working and those not working pre-intervention.

Table 5 indicates that participants were significantly less able to work prior to undertaking the intervention compared to post-intervention and at follow-up, for the overall sample and those working and not working. Pre-intervention, the working group was significantly more productive than the group not working as shown in Figure 1. Post-intervention and follow-up, this difference was no longer significant.

### 3.2. Clinical Scores

**Figure 2:**
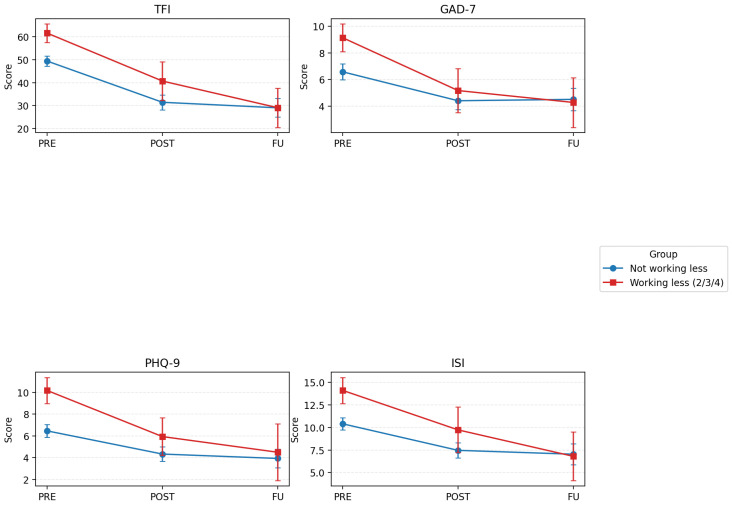
Changes in tinnitus distress (TFI), anxiety (GAD-7), depression (PHQ-9) and insomnia (ISI) pre-intervention (Pre), post-intervention (Post) and at follow-up (FU), comparing those not working less (blue lines) and those working less (due to tinnitus, retiring). Error bars represent 95% CIs.

For all the clinical scores there were significant improvements at post-intervention and follow-up compared to pre-intervention as seen in Figure 2. There were significant group differences for tinnitus distress and depression but not for anxiety and quality of life. There was significant time difference between group interactions for tinnitus, anxiety and insomnia but not for depression and quality of life as shown in Table 6.

### 3.3. Qualitative Analysis

There were 310 participants who provided responses to the open questions. Of these responses, 67 (22%) explained that they have not allowed the tinnitus to interfere with their work and 20 (6%) have found strategies to overcome the tinnitus. The remaining 223 (72%) comments indicated how tinnitus impacted their effectiveness working, as seen in Table 5. The main themes indicating negative effects were disrupted productivity, tinnitus impacting social interactions, reduced work fulfillment, and role changes. A further theme was identified, indicating adaptation strategies to mitigate the effects of tinnitus at work. This also indicated both direct and indirect impacts. Direct impacts included disrupted productivity due to difficulty sustaining attention, slower task completion, not hearing critical information, not meeting the demands and increased errors. Indirect effects encompassed fatigue, withdrawing, higher anxiety and depression, increased frustration and lower work enjoyment as seen in Table 7.

## 4. Discussion

Tinnitus imposes significant healthcare and societal costs [26], highlighting the need for strategies to mitigate its impact. This study examined the effects of tinnitus on workplace functioning, the role of comorbidities, and whether an evidence-based intervention could improve occupational outcomes. During this preliminary study, participants reported higher work productivity at post-intervention and follow-up compared to pre-intervention. Post-intervention improvements were observed in tinnitus distress, anxiety, depression, and insomnia. There was significant time difference between group interactions for tinnitus and anxiety when comparing those working and not working. Qualitative findings highlighted challenges related to attention, fatigue, and communication, as well as coping strategies and role modifications. This study contributes to the tinnitus intervention literature by indicating that undertaking an evidence-based intervention may improve work productivity as outlined in the sections below. This relationship should be further assessed in more robust study designs including a control group.

### 4.1. Impact of Tinnitus on Workplace Functioning

Most participants (81%) reported no change in work patterns due to tinnitus. These results are similar to those found by Coco et al. [8], reporting that 83% of veterans did not report an impact of tinnitus on work. According to the current research, the remaining 11% worked less, 7% stopped working, and 1% received disability allowance. Although global figures for tinnitus-related disability benefits are unavailable, these rates are substantially lower than those observed in the veteran population in the US, where approximately 3.2 million veterans receive disability compensation for tinnitus and 273,502 new claims are approved annually [27]. This discrepancy may reflect established reporting channels within military systems. In contrast, many individuals in civilian settings may avoid disclosing tinnitus-related difficulties due to fear of stigma or discrimination, potentially leading to underreporting and missed career advancement opportunities [28]. The use of items used for workplace productivity and functioning were unvalidated and relied on subjective self-reported perception that could have introduced biased recall. Incorporating validated measures for future studies is required.

In addition to working less because of tinnitus as highlighted above, tinnitus also had impact on work productivity. Due to tinnitus, 11% were working reduced hours, 7% stopped working and 1% were on disability allowance. The present study indicates that more awareness regarding the impact of tinnitus in workplaces is needed to accommodate the challenges faced by these employees and enable them to continue working.

### 4.2. Comorbidities and Reduced Productivity

Qualitative analysis revealed that 22% of participants actively prevented tinnitus from interfering with work, with 6% employing coping strategies. Among those reporting work-related difficulties, tinnitus disrupted productivity by impairing sustained attention, increasing fatigue, complicating work in quiet environments, and causing slower task completion and more errors. Communication challenges were noted, including missed information and social withdrawal. Reduced work fulfillment was linked to heightened anxiety, depression, frustration, and irritability, often resulting in role modifications or career changes. Both direct impacts and indirect impacts were found such as disrupted productivity due to difficulty sustaining attention, slower task completion, not hearing critical information, not meeting the demands and increased errors. Indirect effects encompassed fatigue, withdrawing, higher anxiety and depression, increased frustration and lower work enjoyment. These findings align with previous research indicating that tinnitus-related comorbidities, particularly anxiety and depression, exacerbate occupational challenges [29]. Anxiety and depression are furthermore likely to be the greatest predictors of the impact on daily life from prediction modeling including 21 tinnitus studies [30]. The combination of fatigue, cognitive demands, increased anxiety and depression may all be contributing to reduced productivity.

### 4.3. Effects of Evidence-Based Intervention on Work Productivity

This study was unique in its approach, comparing work productivity before and after undertaking ICBT for tinnitus. The results are considered an indication of possible effects, as only half of the overall sample completed the intervention and post-intervention questionnaires and fewer the 2-month follow-up questionnaire and there was no control group for this study. The preliminary findings indicate that following the intervention, significantly fewer participants reported working reduced hours at both post-intervention and follow-up compared to pre-intervention levels. This improvement was observed in the overall sample and among those who were employed. In addition, tinnitus-related distress and comorbidities, including anxiety, depression, and quality of life, showed significant improvements at post-intervention and follow-up relative to baseline scores. Moreover, qualitative feedback supported these findings, with participants indicating that the intervention enhanced their ability to work. Reduced reports of working less after the intervention suggest that targeted tinnitus management may mitigate occupational limitations. These results may not be fully directly associated with undertaking the intervention as they did not account for various possible confounding variables such as whether participants received concurrent tinnitus-related treatments during the study period or whether or not there were changes to their work environment or other life stressors. Further studies need to adjust for these possible confounding variables.

### 4.4. Policy Implications

Existing policies and legal frameworks address hearing loss through noise exposure regulations and disability legislation. Within these frameworks, tinnitus should be explicitly recognized as a potential disability. Standardized workplace policies are needed to ensure employees with tinnitus can access reasonable adjustments, such as strategies to improve concentration (e.g., access to tinnitus maskers, or specialized headphones or headsets) and reduce cognitive load. Such adjustments may include flexible scheduling, social support, access to healthcare, optimized workspaces, and assistive listening devices to improve overall functionality. A large proportion of individuals with tinnitus also have hearing loss and may benefit from the use of hearing aids in the workplace. Ways to improve the availability of tinnitus interventions should furthermore be sought by employees. Employer education is furthermore critical to reduce stigma and foster supportive environments [6].

### 4.5. Study Limitations and Future Directions

While the study highlighted the impact of tinnitus on work functioning and more importantly the effect of intervention, the study had a few limitations. The lack of a control or comparison group makes it difficult to determine whether the observed improvements are attributable to the specific effects of ICBT, natural change over time, placebo effects, or regression to the mean. The study relied on self-reported perception that could introduce recall bias and desirability bias. Furthermore, many questions such as ‘Do you work less due to having tinnitus’, lacked established validity and reliability evidence.

There was potential sampling bias due to the fact that there was a large number of people who failed to fill in the post-intervention questionnaires, and it could be that only those who experienced positive changes were inclined to fill them in. There were numerous reasons provided for the attrition identified, such as time-constraint, participants having competing life priorities such as caregiving responsibilities, or disengaging either after receiving enough benefit or finding the questionnaire too onerous. There may be other potential confounding variables that may have affected the results that were not accounted for such as whether participants received concurrent tinnitus-related treatments during the study period, changes in the work environment or change in work environments or demands. Moreover, live events and/or stressors may furthermore affect work productivity independently of the intervention. Future studies need to address the confounding variables and current study limitations.

Addressing the cognitive load associated with tinnitus is essential for improving work productivity. The Job Demands–Resources Model [31] offers a useful framework for examining how job demands, such as workload, time pressure, emotional strain, and role ambiguity, interact with resources to influence outcomes. According to the Job Demands–Resources Model, sufficient resources can buffer the negative impact of high demands, enhancing engagement and productivity, whereas low resources combined with high demands increase the risk of burnout and absenteeism. This model could be incorporated during future research. More comorbidities can be included, such as cognitive difficulties, hearing loss, hyperacusis and various combinations of these. The focus on the impact of tinnitus on different types of occupational needs could also be explored to provide job role modifications. Research can also explore workplace interventions that reduce cognitive and emotional strain, including evidence-based approaches such as CBT, sound therapy, and hearing aids [32]. Implementing standardized reporting systems, workplace policies, and procedures could help identify and mitigate these effects. Digital health tools and integrated audiological and occupational health protocols could furthermore be advocated to improve addressing the difficulties those with tinnitus may face in the workplace.

## 5. Conclusions

This study illustrates possible ways tinnitus can adversely affect work performance and productivity, both directly and indirectly. Undertaking an evidence-based intervention for tinnitus can mitigate these effects and also reduce tinnitus distress and the associated anxiety, depression and insomnia. Supporting employees in accessing tinnitus interventions may be particularly beneficial, given the significant improvements observed in this study.

## Figures and Tables

**Table 1:** Internet-based intervention for tinnitus program overview.

Week	Cognitive Behavioral Therapy Techniques	Progressive Relaxation Guide	Dealing with the Effects of Tinnitus
1	Introduction to the program Understanding tinnitus	Deep relaxation	Sound enrichment
2	Positive imagery	Deep breathing	Sleep guidelines
3	Reinterpretation of tinnitus	Entire body relaxation	Improving focus
4	Shift focus away from tinnitus	Frequent relaxation	Increasing sound tolerance
5	Thought pattern identification	Quick relaxation	Communication tactics
6	Challenging thoughts (Cognitive restructuring)	Incorporating a relaxation routine	Summary overview of the program
7	Being mindful	Implementing the designed relaxation routine	Future planning to maintain the tinnitus effects
8	Exposure to tinnitus	Evaluating the effects	Evaluating the intervention effects

**Table 2:** Clinical characteristics.

Characteristics	Full Sample (*n* = 449)	Those Undertaking the Intervention and Completing Post-Intervention Scores (*n* = 200)	Not Working Less (*n* = 285)	Working Less (*n* = 164)	Group Differences (Working and Not Working)
Age mean in years (SD)	54.4 (13.4)	56.7 (12:12)	50.0 (11.6)	62.0 (SD: 13.0)	*t*(447) = −10.149, *p* = 0.001
Gender Male Female	219 (49%) 230 (51%)	118 (59%) 82 (41%)	152 (34%) 133 (30%)	67 (15%) 97 (21%)	*X*^2^ (1) = 4.49, *p* = 0.01
Ethnicity Hispanic or Latino Non- Hispanic	96 (21%) 353 (78.6%)	37 (19%) 163 (81%)	73 (16%) 212 (48%)	23 (5%) 141 (31%)	*X*^2^ (1) = 8.32, *p* = 0.004
Race American Indian Asian Pacific Islander Black White More than one race	5 (1%) 9 (2%) 1 (0.2%) 9 (2%) 396 (88%) 29 (6.8%)	3 (1.5%) 2 (1%) 1 (0.5%) 5 (2.5%) 182 (91%) 7 (3.5%)	3 (0.7%) 7 (1.6%) 0 4 (0.9%) 247 (55%) 24 (5.3%)	2 (0.4%) 2 (0.4%) 1 (0.2%) 5 (1.1%) 149 (33.2%) 5 (1.1%)	*X*^2^ (5) = 8.82, *p* = 0.116
Tinnitus duration mean in years (SD)	12 (13.1)	13 (14.44)	12.2 (13.5)	11.6 (12.3)	*t*(447) = 0.51, *p* = 0.61
Undertaking the tinnitus Intervention completing the post-intervention scores Completing 2-month follow-up scores	180 (40%) 122 (27%)		109 (24%) 77 (17%)	71 (16%) 45 (10%)	

SD: Standard deviation.

**Table 3:** Comparing those completing the post-intervention questionnaire and those not completing this questionnaire.

Characteristics	Full Sample (*n* = 449)	Those Completing the Post-Intervention Questionnaire (*n* = 200)	Those NOT Completing the Post-Intervention Questionnaire (*n* = 249)	Group Differences (Completing and Not Completing)
Age mean in years (SD)	54.4 (13.4)	57 (12.1)	52 (15.1)	
Gender Male Female	219 (49%) 230 (51%)	82 (41%) 118 (59%)	137 (55%) 112 (45%)	*X*^2^ (1) = 8.17, *p* = 0.004
Tinnitus duration mean in years (SD)	12 (13.1)	13.1 (14.4)	11.7 (13.3)	*X*^2^ (5) = 8.82, *p* = 0.12
TFI mean scores Pre- intervention	52.5 (SD: 21.6)	54.11 (SD: 20.73)	51.15 (SD: 22.27)	*t*(447) = 1.44, *p* = 0.15
GAD-7 mean scores Pre- intervention	7.21 (SD: 5.65)	7.20 (SD: 5.43)	7.22 (SD: 5.85)	*t*(447) = −0.03, *p* = 0.97
PHQ-9 mean scores Pre-intervention	7.38 (SD: 5.94)	7.37 (SD: 5.73)	7.39 (SD: 6.13)	*t*(447) = −0.03, *p* = 0.98
ISI mean scores Pre-intervention	11.3 (SD 6.7)	11.69 (SD: 6.53)	10.96 (SD: 6.90)	*t*(447) = 1.12, *p* = 0.26
EQ 5D VAS mean score Pre-intervention	74.1 (SD: 15.9)	75.09 (SD:15.46)	73.29 (SD: 16.17)	*t*(447) = 1.18, *p* = 0.24

TFI = Tinnitus Functional Index, GAD 7 = Generalized Anxiety Disorder. PHQ-9 = Patient Health Questionnaire 9; ISI= The insomnia-severity index; EQ-5D-5 = Euroqol health-related quality of life questionnaire; SD: standard deviation. Participants indicating that tinnitus was the reason for working less at pre-intervention, post-intervention and at follow-up is seen in Table 4.

**Table 4:** Participants indicating that tinnitus was the reason for working less at pre-intervention, post-intervention and at follow-up.

Pre-intervention	Full Sample (*n* = 401)	Not Working Less (*n* = 257)	Not Working or Reduced Working (*n* = 144)	Between Group Significance
Not working less Reduce hours Stopped work Disability allowance	325 (81%) 42 (11%) 27 (7%) 7 (1%)	221 (55%) 33 (8%) 2 (0.5%) 1 (0.2%)	104 (26%) 9 (2%) 25 (6%) 6 (2%)	*X*^2^ (3) = 51.2, *p* < 0.001
**Post-intervention**	**Full sample (*n* = 180)**	**Working (*n* = 109)**	**Not working (*n* = 71)**	**Significance**
Not working less Reduce hours Stopped work Disability allowance	163 (91%) 4 (2%) 11 (6%) 2 (1%)	104 (58%) 1 (1%) 3 (2%) 1 (1%)	59 (33%) 3 (2%) 8 (4%) 1 (1%)	*X*^2^ (3) = 8.03, *p* = 0.045
**2-month follow-up**	**Full sample (*n* = 122)**	**Working (*n* = 77)**	**Not working (*n* = 45)**	**Significance**
Not working less Reduce hours Stopped work Disability allowance	108 (89%) 4 (3%) 9 (7%) 1 (1%)	73 (60%) 2 (2%) 2 (2%) 0	35 (29%) 2 (2%) 7 (6%) 1 (1%)	*X*^2^ (3) = 9.40, *p* = 0.024
**Between Time period significance**	*X*^2^ (703, *df* = 4) = 17.42, *p* = 0.008	*X*^2^ (443, *df* = 4) = 20.96, *p* = 0.002	*X*^2^ (260, *df* = 4) = 3.66, *p* = 0.722	

**Table 5:** Participants indicating that tinnitus was the reason for working less effectively pre-intervention, post-intervention and at follow-up.

Preintervention	Full Sample (*n* = 401)	Not Working Less (*n* = 257)	Not Working or Reduced Working Due to Tinnitus (*n* = 114)	Significance (Between the Groups)
Not less effective Slightly less effective Considerably less effective Very much less effective	171 (43%) 177 (44%) 35 (9%) 18 (4.5%)	103 (26%) 129 (32%) 19 (5%) 6 (2%)	68 (17%) 48 (12%) 16 (4%) 12 (3%)	*X*^2^ (3) = 5.9, *p* = 0.001
**Post-intervention**	**Full** **sample (*n* = 180)**	**Not working less (*n* = 109)**	**Not working or reduced working (*n* = 71)**	**Significance**
Not less effective Slightly less effective Considerably less effective Very much less effective	115 (64%) 54 (30%) 8 (4%) 3 (2%)	70 (39%) 34 (19%) 3 (2%) 2 (1%)	45 (25%) 20 (11%) 5 (3%) 1 (1%)	*X*^2^ (3) = 1.96, *p* = 0.580
**2-month follow-up**	**Full #sample (*n* = 122)**	**Not working less (*n* = 77)**	**Not working or reduced working (*n* = 45)**	**Significance**
Not less effective Slightly less effective Considerably less effective Very much less effective	91 (75%) 22 (18%) 6 (5%) 3 (2%)	54 (44%) 17 (14%) 3 (3%) 3 (3%)	37 (30%) 5 (4%) 3 (3%) 0	*X*^2^ (3) = 4.65, *p* = 0.20
**Significance (within-group at different time points)**	*X*^2^ (4) = 50.13, *p* = 0.001	*X*^2^ (4) = 443, *p* = 0.001	*X*^2^ (4) = 260, *p* = 0.002	

**Table 6:** Comparison of the clinical scores of those working and not working at pre-intervention, post-intervention and at follow-up.

Characteristics	Full Sample (*n* = 436 Pre, 200 Post and 132 Follow-Up)	Not Working Less	Working Less or Not Working	Repeated Measures Statistical Results
TFI mean scores Pre- intervention Post- intervention 2-month follow-up	52.5 (SD: 21.6) 33.1 (SD: 22.2) 29.1 (SD: 22.4)	52.2 (SD: 20.8) 33.60 (SD: 22.1) 30.1 (SD: 23.4)	53.0 (SD: 23.0) 32.1 (SD: 22.6) 27.4 (SD: 20.7)	Time F(2, 266) = 157.63; *p* = 0.001 Group: F(1, 266) = 6.00; *p* = 0.014 Time × Group: F(2, 266) = 7.70; *p* = 0.001
GAD-7 mean scores Pre- intervention Post- intervention 2-months follow-up	7.21 (SD: 5.65) 4.55 (SD: 4.44) 4.48 (SD: 4.57)	7.38 (SD: 5.46) 4.70 (SD: 4.15) 4.60 (SD: 4.89)	6.93 (SD: 5.99) 4.30 (SD: 4.89) 4.28 (SD: 4.04)	Time: F(2, 260) = 30.54; *p* = 0.001 Group: F(1, 260) = 0.10; *p* = 0.749 Time × Group: F(2, 260) = 4.00; *p* = 0.02
PHQ-9 mean scores Pre-intervention Post- intervention 2-months follow-up	7.38 (SD: 5.94) 4.51 (SD: 4. 56) 4.01 (SD: 4.82)	7.22 (SD: 5.70) 4.53 (SD: 4.23) 3.81 (SD: 4.28)	7.65 (SD: 6.34) 4.72 (SD: 5.05) 4.34 (SD: 5.58)	Time: F(2, 254) = 34.75; *p* = 0.001 Group: F(1, 254) = 4.75; *p* = 0.0303 Time × Group: F(2, 254) = 1.83; *p* = 0.163
ISI mean scores Pre-intervention Post- intervention 2-months follow-up	11.3 (SD 6.7) 7.85 (SD: 5.94) 7.0 (SD: 6.24)	10.4 (SD: 6.21) 7.46 (SD: 5.54) 7.04 (SD: 6.29)	14.1 (SD: 7.51) 9.73 (SD:7.4) 6.8 (SD 6.1)	Time: F(2, 244) = 64.71; *p* = 0.001 Group: F(1, 244) = 12.31; *p* = 0.001 Time × Group: F(2, 244) = 6.17; *p* = 0.002
EQ 5D VAS mean score Pre-intervention Post- intervention 2-months follow-up	74.1 (SD: 15.9) 77.9 (SD 15.1) 80.1 (SD: 14.8)	75.2 (SD: 14.7) 77.5 (SD: 15.7) 80.9 (SD: 15.5)	72.2 (SD: 17.5) 78.4 (SD: 14.1) 79.0 (SD: 13.7)	Time: F(1, 129) = 5.41; *p* = 0.022 Group: F(1, 129) = 0.52; *p* = 0.471 Time × Group: F(1, 129) = 0.24; *p* = 0.624

TFI = Tinnitus Functional Index, GAD 7 = Generalized Anxiety Disorder. PHQ-9 = Patient Health Questionnaire 9; ISI = The insomnia-severity index;EQ-5D-5 = Euroqol health-related quality of life questionnaire; SD = standard deviation.

**Table 7:** Qualitative analysis of open-ended questions related to work.

Category	Sub-Category	No of Meaning Unit	Examples
Disrupted Productivity	Sustaining attention	29	Due to the tinnitus, I now have trouble reading and concentrating. To comprehend what is written, I have to reread, over and over again. Days of high tinnitus I am rarely able to focus at work.
	Fatigue	25	I had to miss work yesterday due to lack of sleep-was a zombie and not functional.
	Difficulty working in quiet environments	20	It is difficult sitting at a computer, working with no other sounds around
	Increased errors and less productivity	14	When very loud. I cannot concentrate and make mistakes. It causes me to get distracted so I am not as productive as I could be.
	Slower task completion	12	I have had to work many more later hours to accomplish deadlines, and this is because of the tinnitus distracting my clarity of thought and ability to be continuous in the story I’m writing. I’ve just been pushing through it. I worry that it will affect my job and my pay.
Impacting interactions	Not hearing crucial information	24	I interview students and sit in meetings. It’s very hard to hear students’ answers. especially if they are soft spoken. It’s also difficult to hear in meetings. I cannot talk on the phone the noise is making it hard to understand and hear people talk.
	Withdrawing	11	I cancel meetings due to the tinnitus. It affects my ability to communicate in group situations. So, where possible I spread out or reduce my group activities, so they aren’t to frequent.
Reduced work fulfillment	Increased anxiety/depression	8	I got fired from work as due to the tinnitus I was always anxious and depressed which lowered my productivity.
	Frustration and irritability	7	I get irritable easily, so I only do necessary tasks. I am around loud sounds because of my job. The ringing after-effects linger for the rest of the evening.
	Reduced enjoyment of work	11	I primarily volunteer with children; it used to be more enjoyable—but now the loudness and being unable to hear soft voices makes me feel less effective and have less joy in what I am doing.
Role changes	Modifications	16	I’ve had to change from working with clients to an administrative role
	Career shift	12	I had to quit working with a band because I don’t go where it’s very loud. I can’t attend big public events.
	Unable to meet the demands	9	I retired early due to complications concentrating. I was an operator on a DCS and could no longer hear some alarms or understand the radio. Which was getting very dangerous for others.
Adaptation strategies	Utilizing strategies	13	I’ve learned to focus on the activity at hand rather than the tinnitus.
	Overcoming challenges	7	When I first got tinnitus, it was a problem at work. Now it bothers me sometimes, but I can push my way through it.
	Not interfering	60	No, it hasn’t. It’s in my attitude. I won’t let it get in my way at work. I won’t let it diminish my performance.

## Data Availability

The original data presented in the study are openly available in FigShare at https://doi.org/10.6084/m9.figshare.311645321 (accessed on 25 January 2026).

## Abbreviations

The following abbreviations are used in this manuscript:

ICBT	Internet-based cognitive behavioral therapy intervention
ISI	insomnia-severity index
EQ-5D-5L	Health-related quality of life (Euroqol)
TFI	Tinnitus Functional Index
GAD7	Generalized Anxiety Disorder Questionnaire
PHQ9	Patient Health Questionnaire 9
